# Supplementary material for: Alicyclic β- and γ-Amino Acids: Useful Scaffolds for the Stereocontrolled Access to Amino Acid-Based Carbocyclic Nucleoside Analogs
Source: Molecules. 2019 Jan 3;24(1):161. doi: 10.3390/molecules24010161 (PMC6337571; doi:10.3390/molecules24010161)
Supplement: Supplementary file 1 [file molecules-24-00161-s001.pdf]

Supplementary Materials:

Alicyclic β- and γ-amino acids: useful scaffolds for the stereocontrolled access to amino acid-based carbocyclic nucleoside analogues

Attila Márió Remete,[1,2] Loránd Kiss[1,2*]

[1]Institute of Pharmaceutical Chemistry, University of Szeged, H-6720 Szeged, Eötvös u. 6, Hungary

[2]University of Szeged, Interdisciplinary Excellence Centre, Institute of Pharmaceutical Chemistry

E-mail: kiss.lorand@pharm.u-szeged.hu; kiss.lorand00@gmail.com;

web: http://www2.pharm.u-szeged.hu/gyki

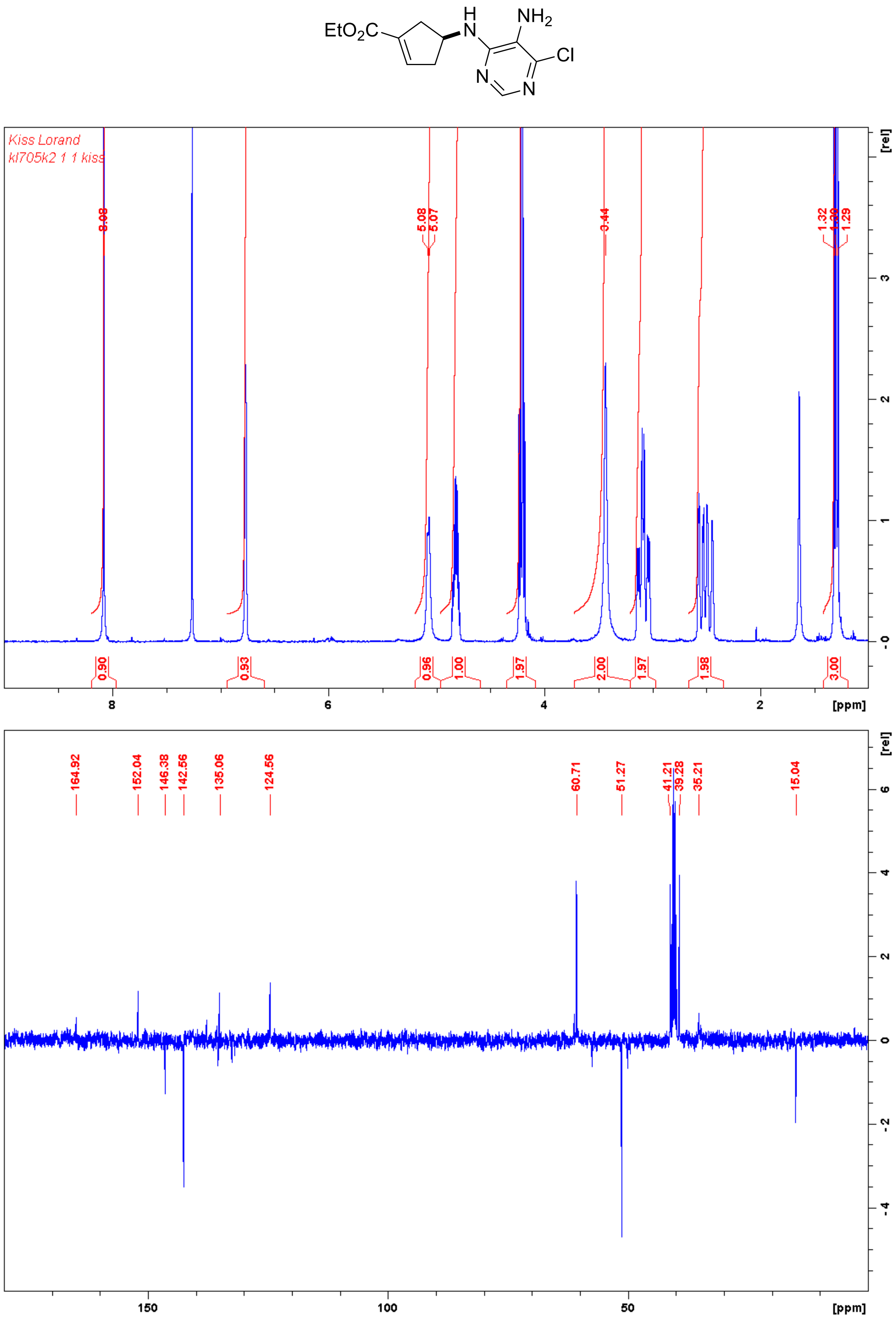

Figure 1. ^{1}H-NMR and ^{13}C-NMR spectra of compound **(±)-16**

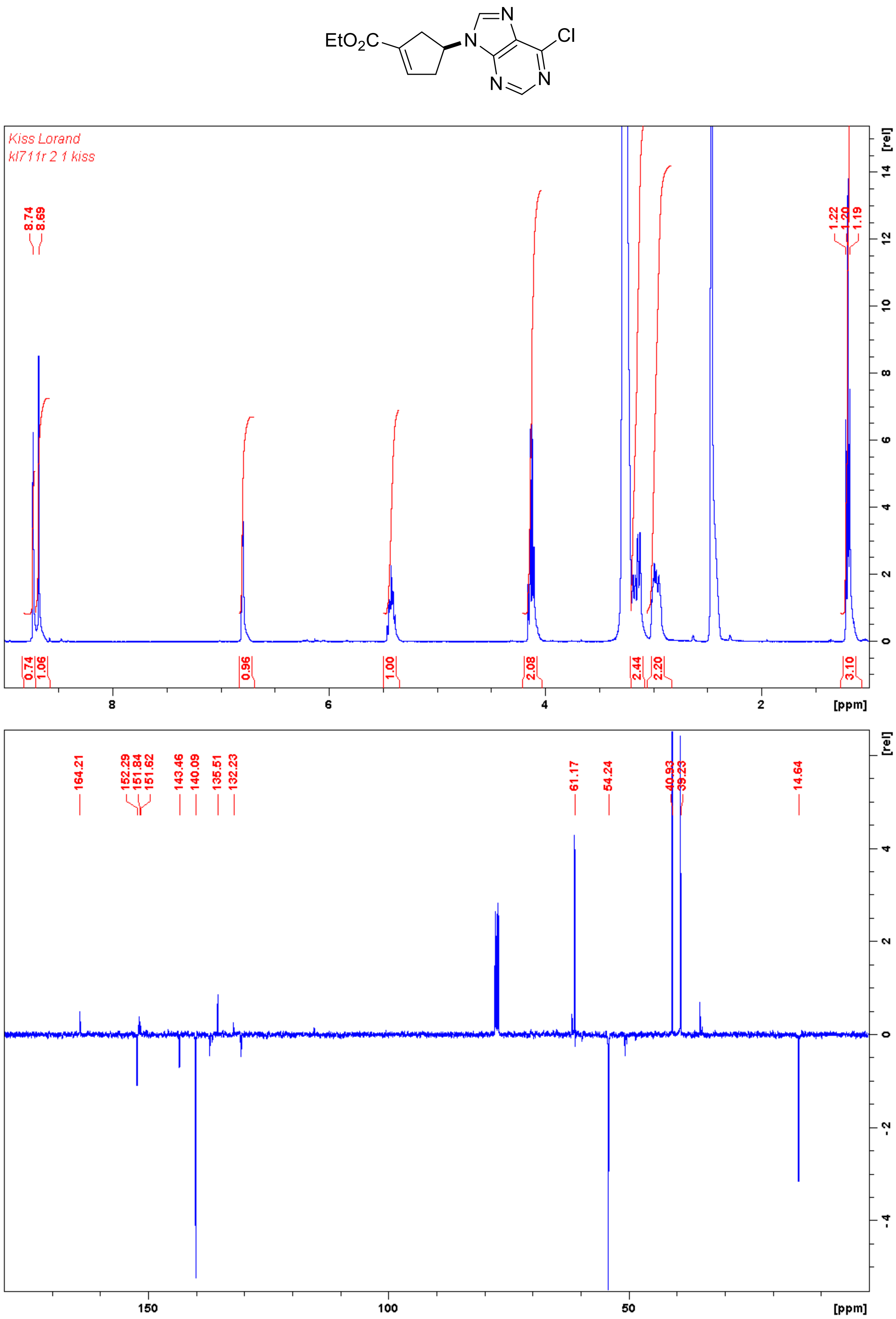

Figure 2. ^{1}H-NMR and ^{13}C-NMR spectra of compound **(±)-17**

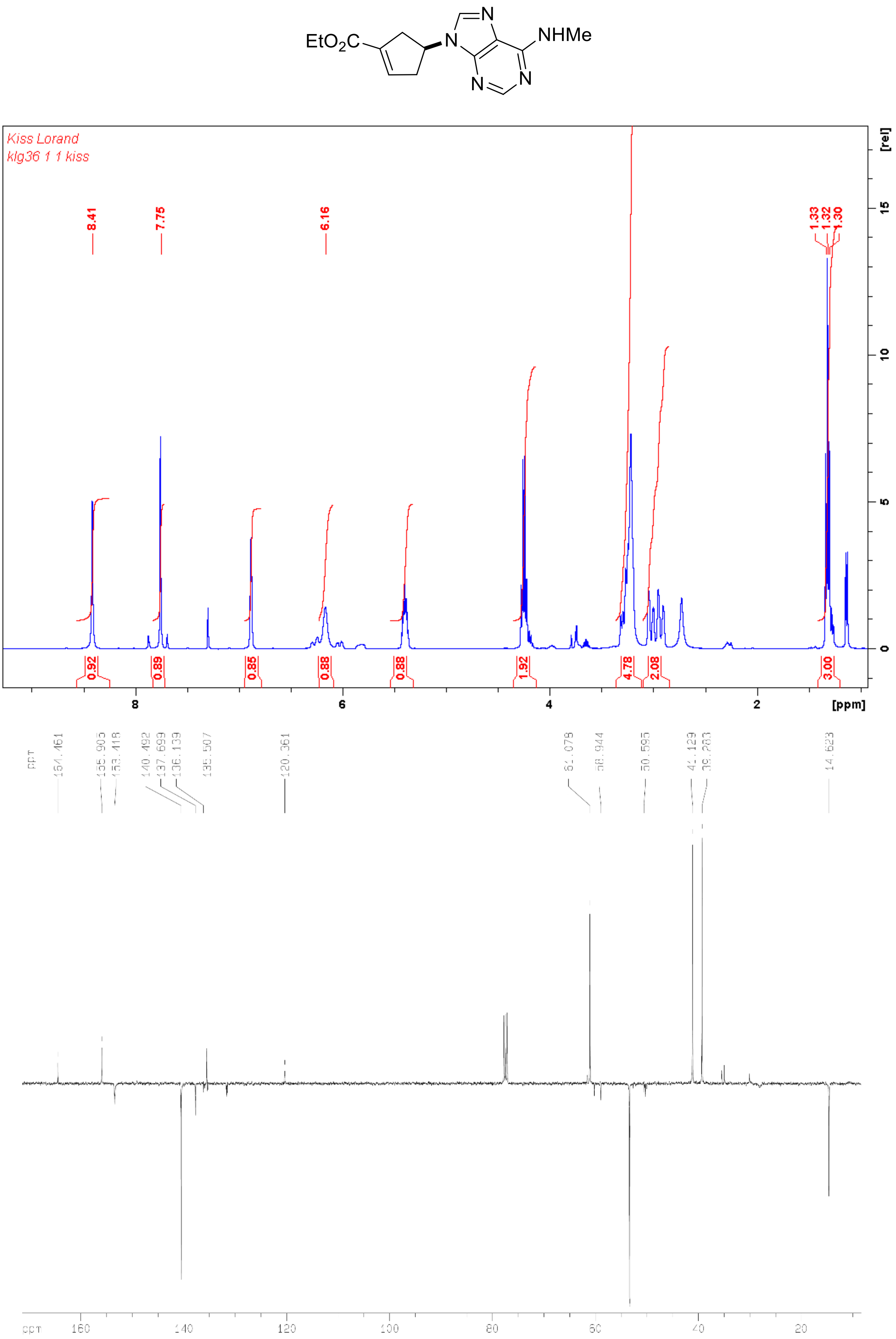

Figure 3. ^{1}H-NMR and ^{13}C-NMR spectra of compound **(±)-18**

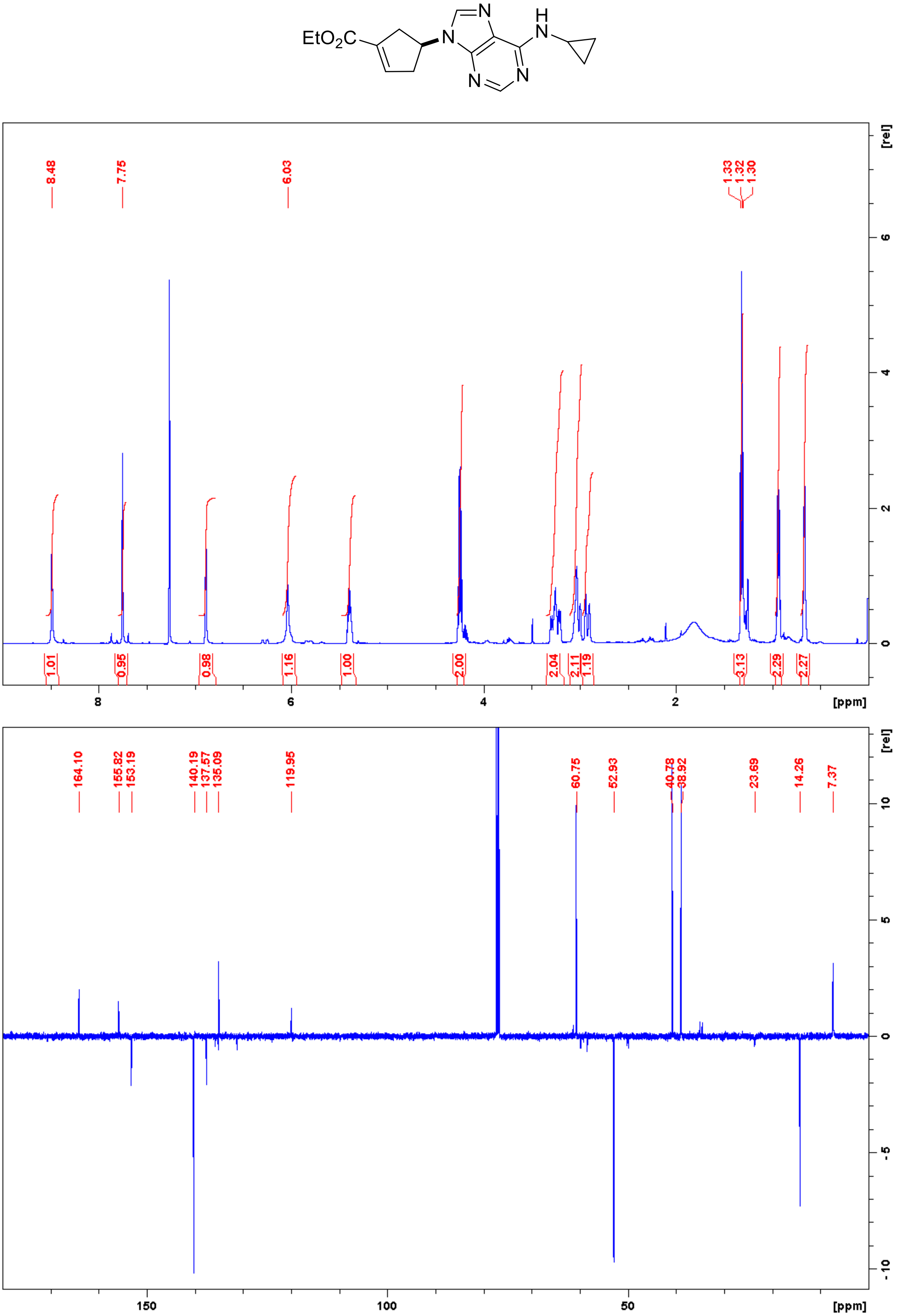

Figure 4. ^{1}H-NMR and ^{13}C-NMR spectra of compound **(±)-19**

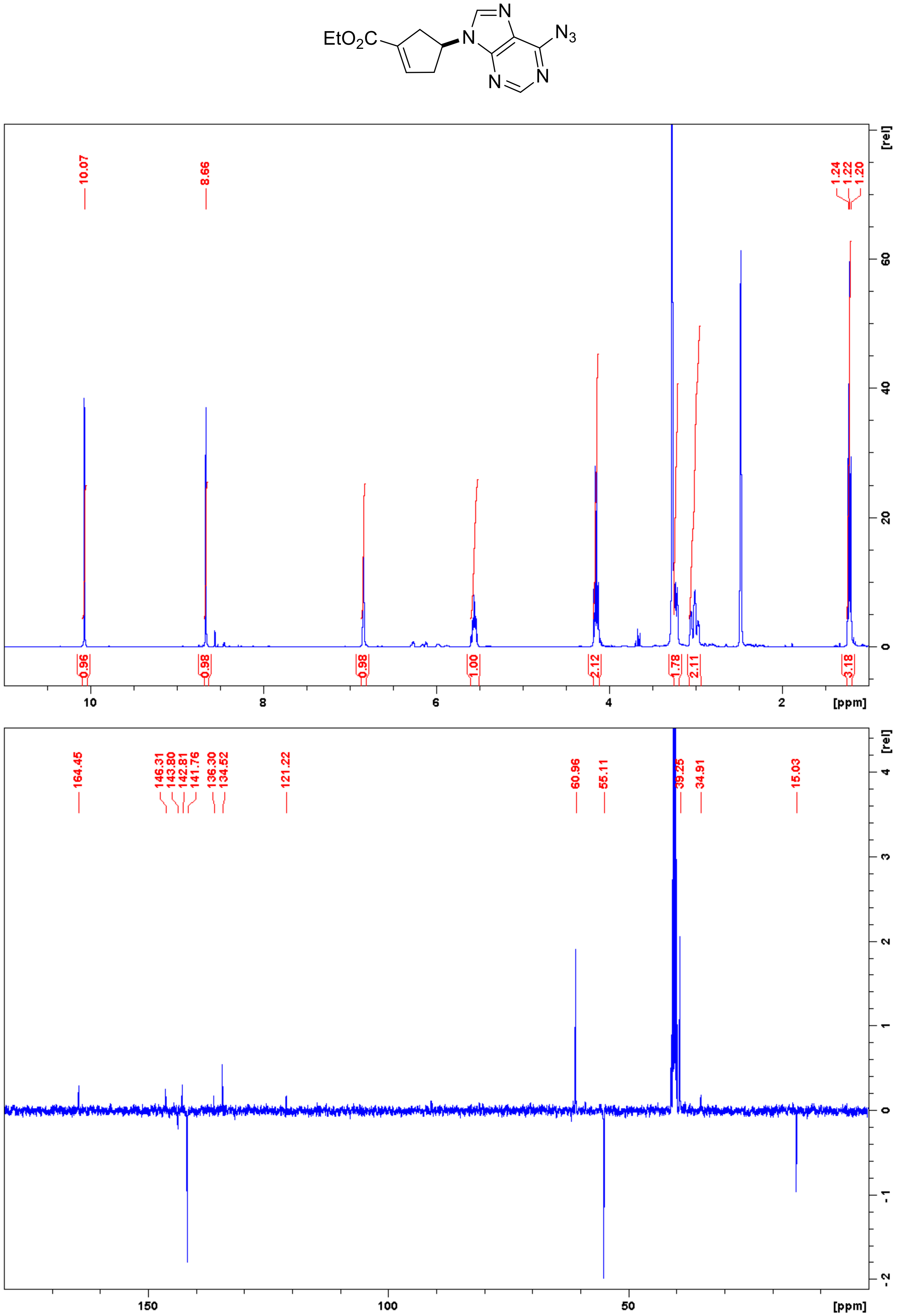

Figure 5. ^{1}H-NMR and ^{13}C-NMR spectra of compound **(±)-20**

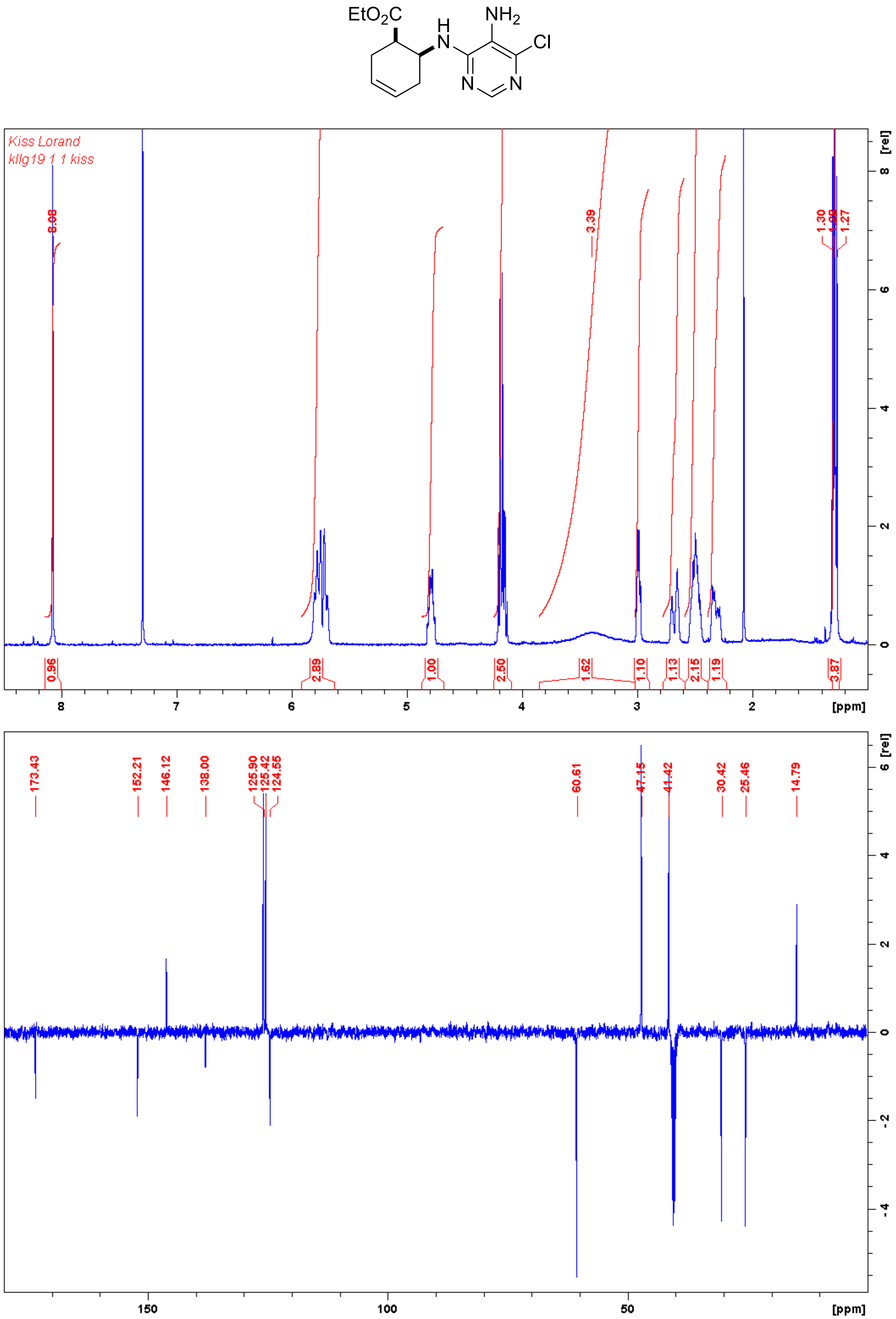

Figure 6. ¹H-NMR and ¹³C-NMR spectra of compound **(±)-23**

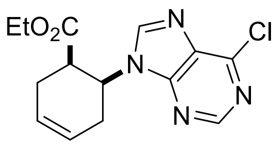

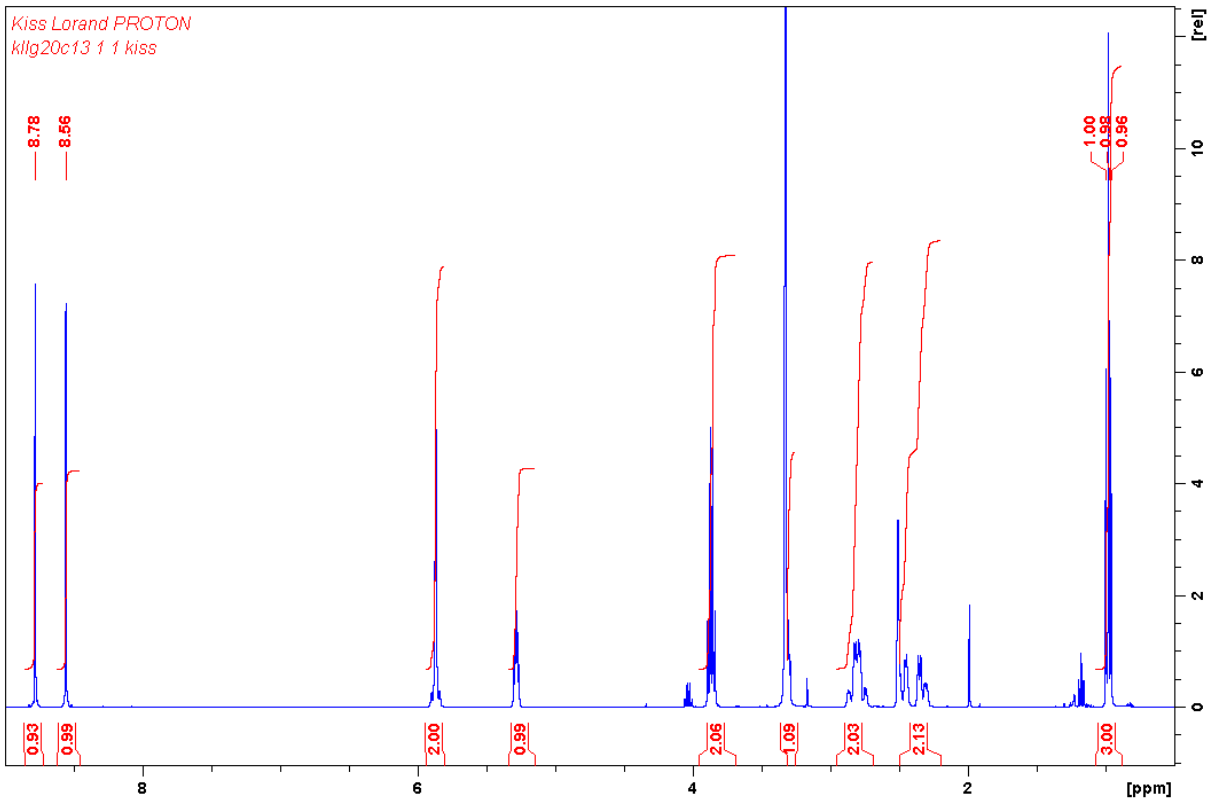

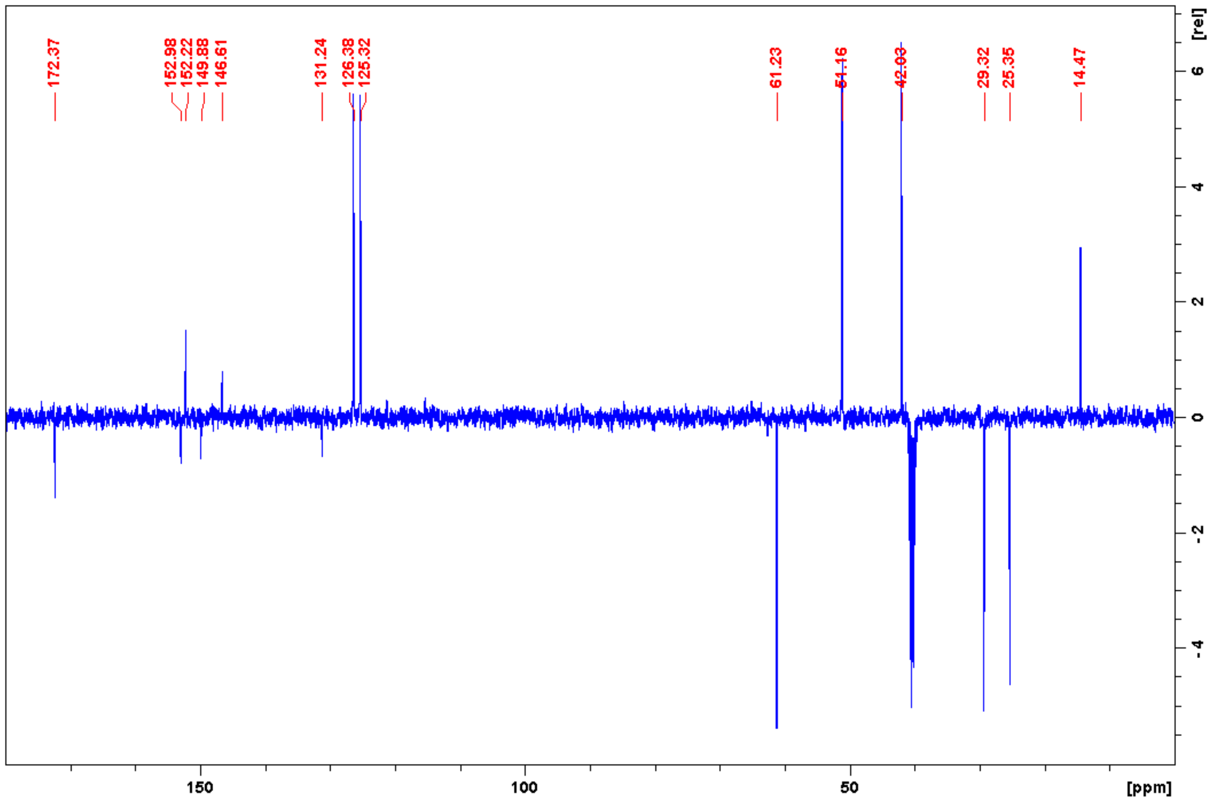

Figure 7. ^{1}H-NMR and ^{13}C-NMR spectra of compound **(±)-24**

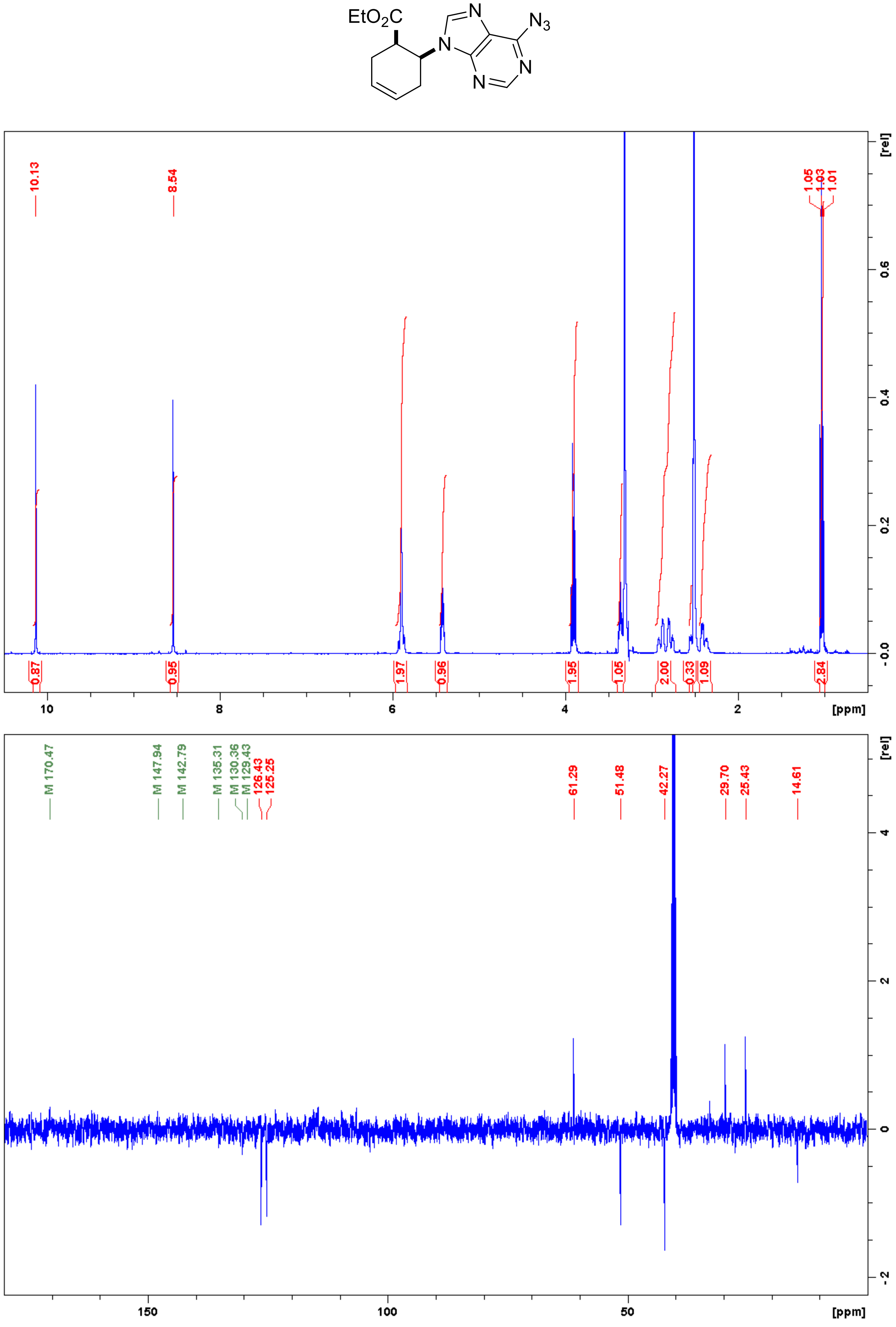

Figure 8. ^{1}H-NMR and ^{13}C-NMR spectra of compound **(±)-25**

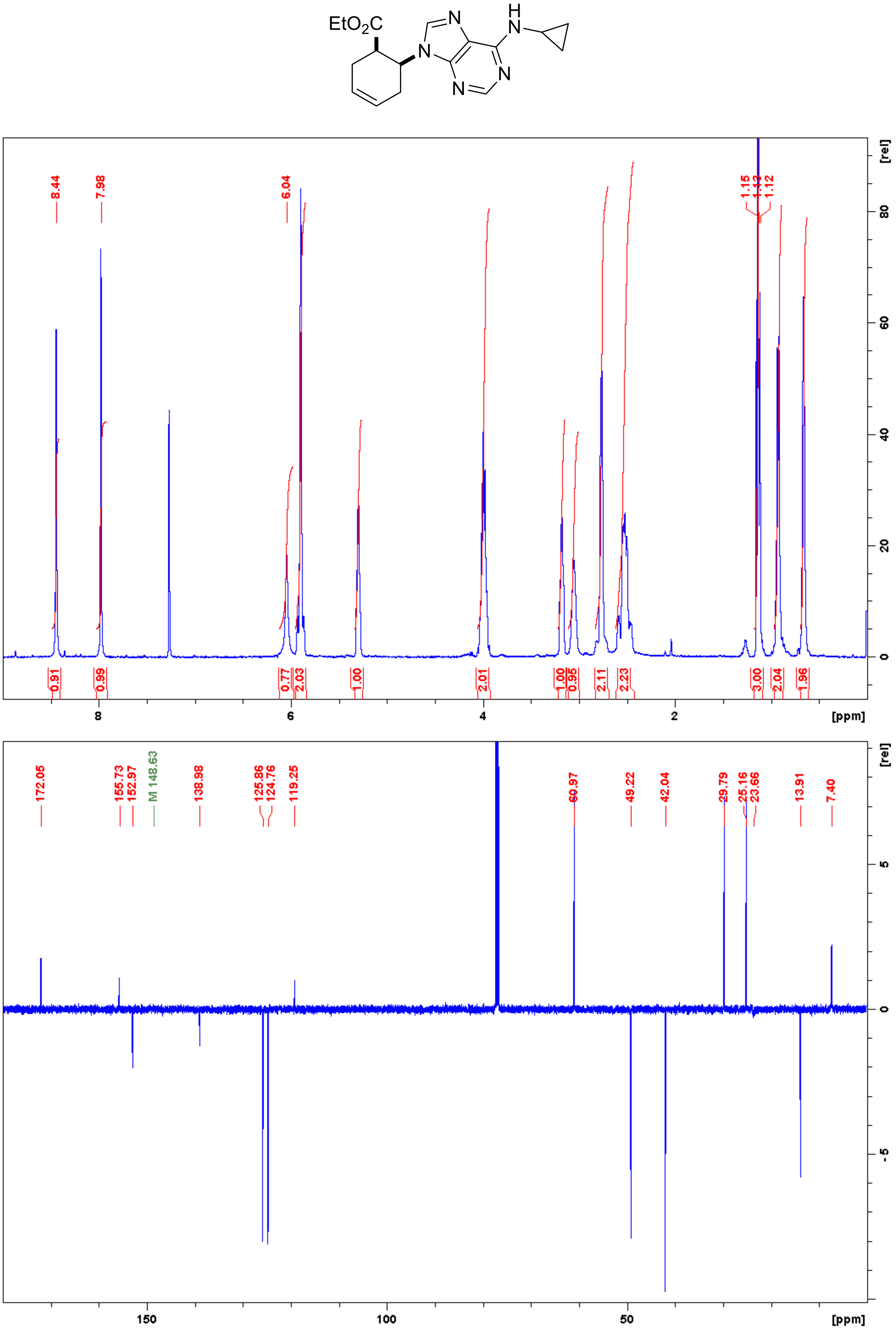

Figure 9. ^{1}H-NMR and ^{13}C-NMR spectra of compound **(±)-26**

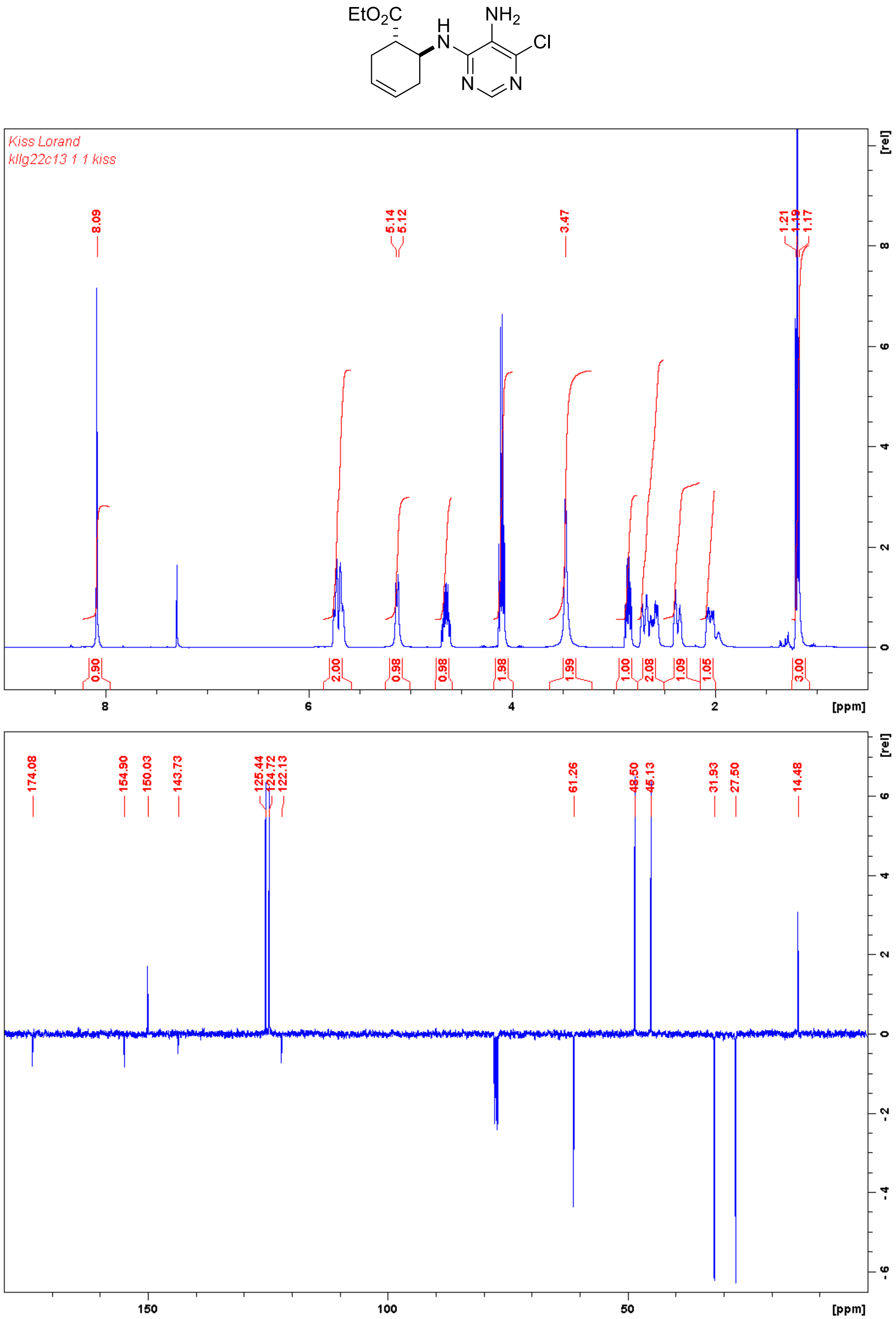

Figure 10. ^{1}H-NMR and ^{13}C-NMR spectra of compound **(±)-29**

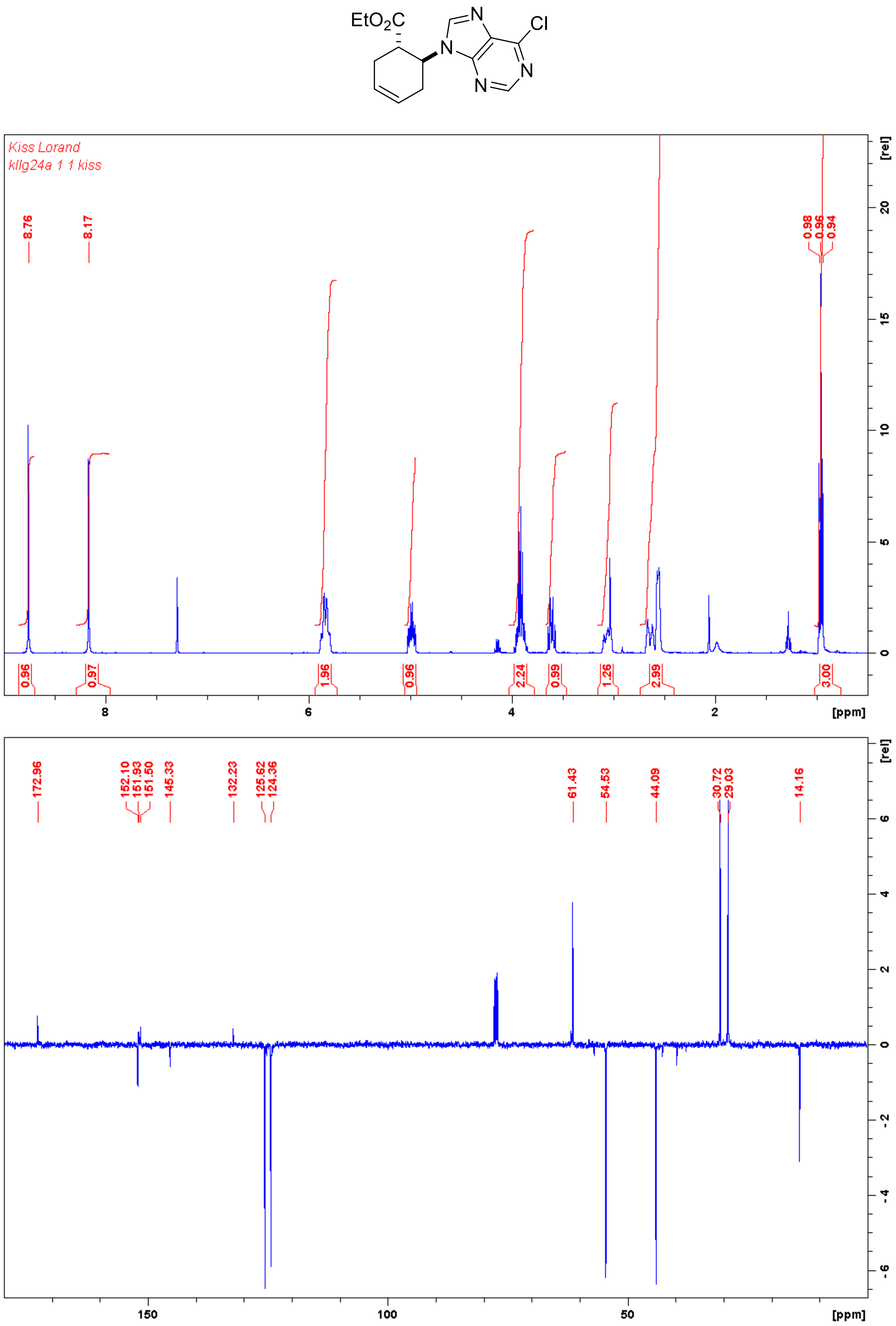

Figure 11. ^{1}H-NMR and ^{13}C-NMR spectra of compound **(±)-30**

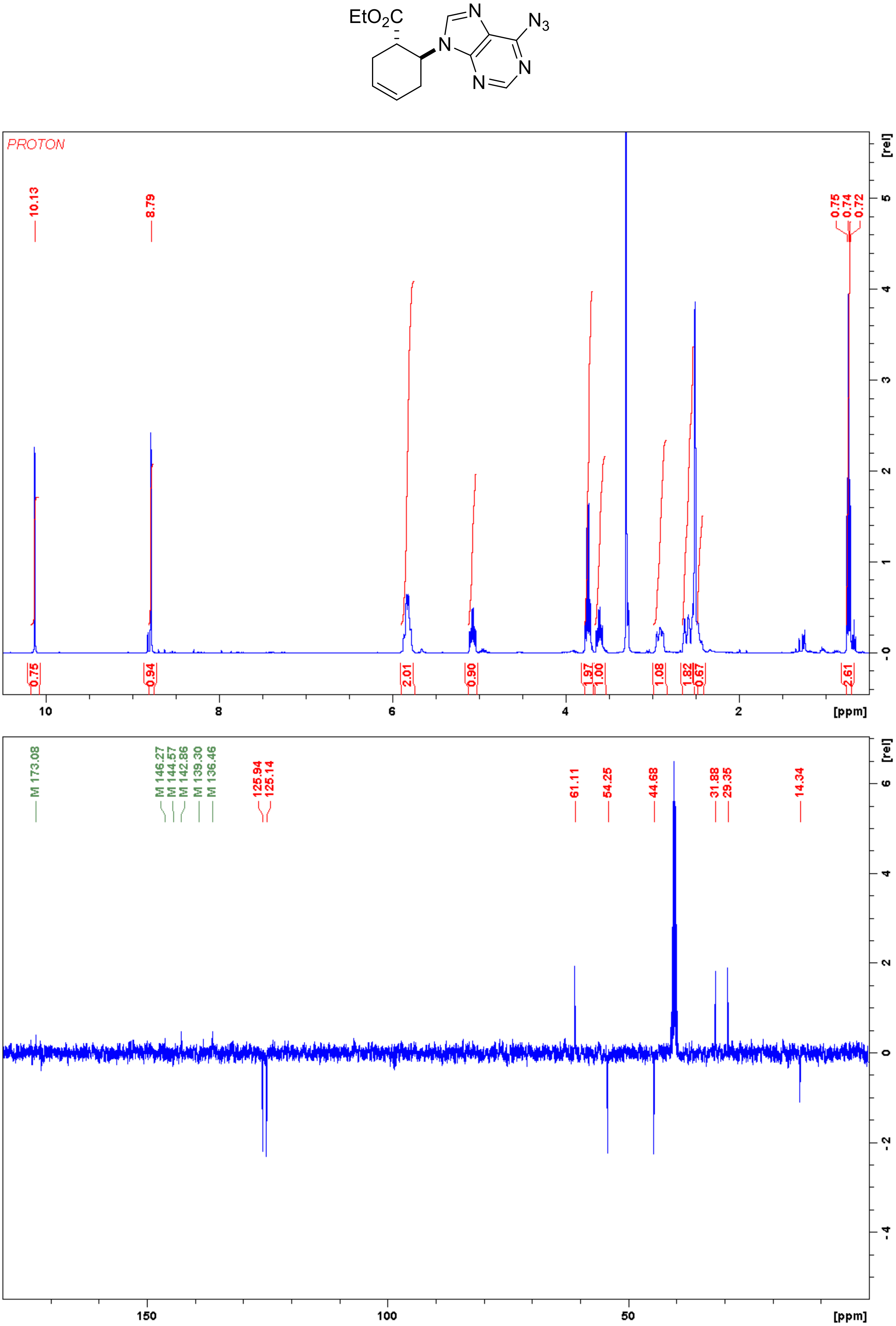

Figure 12. ^{1}H-NMR and ^{13}C-NMR spectra of compound **(±)-31**

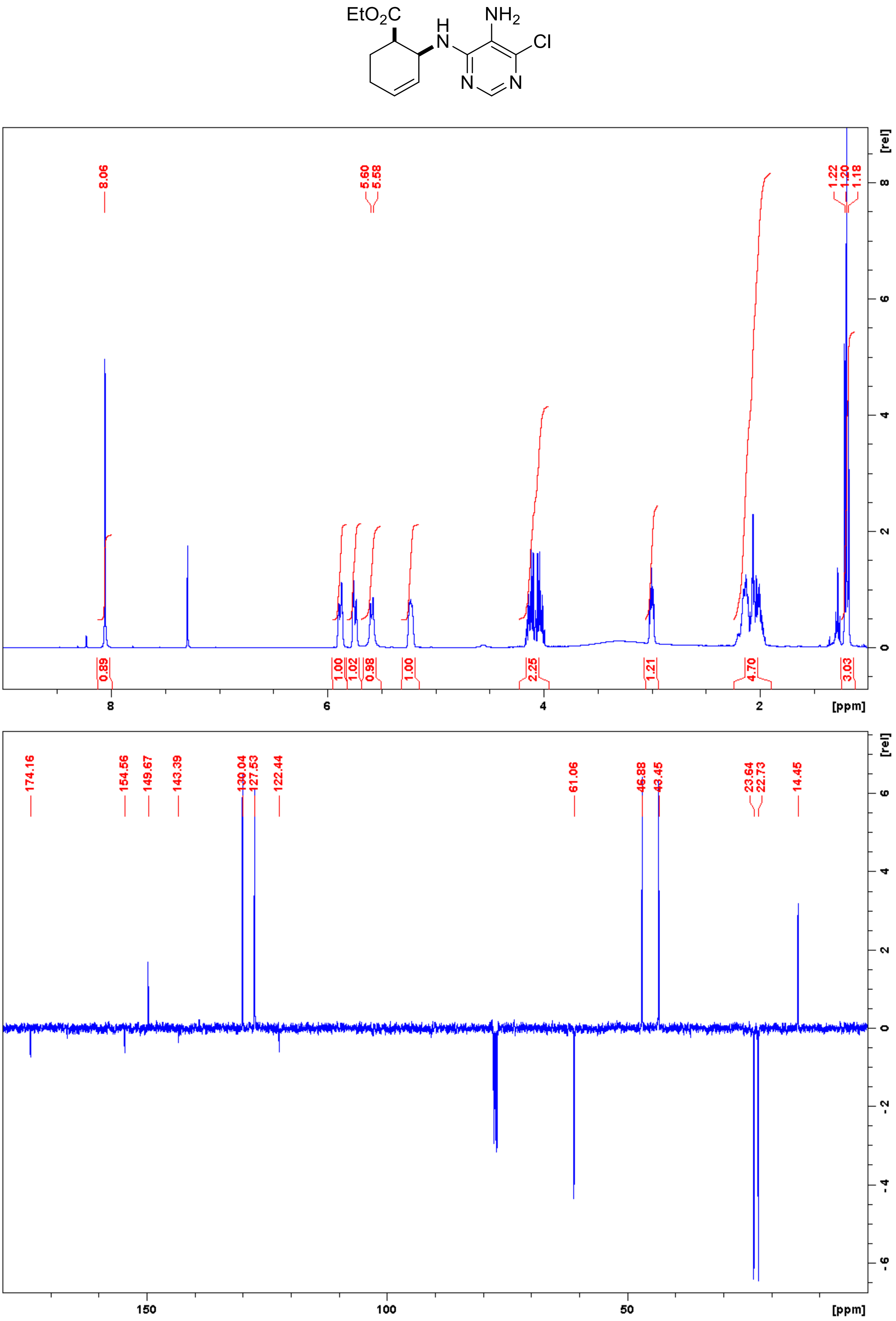

Figure 13. ^{1}H-NMR and ^{13}C-NMR spectra of compound **(±)-34**

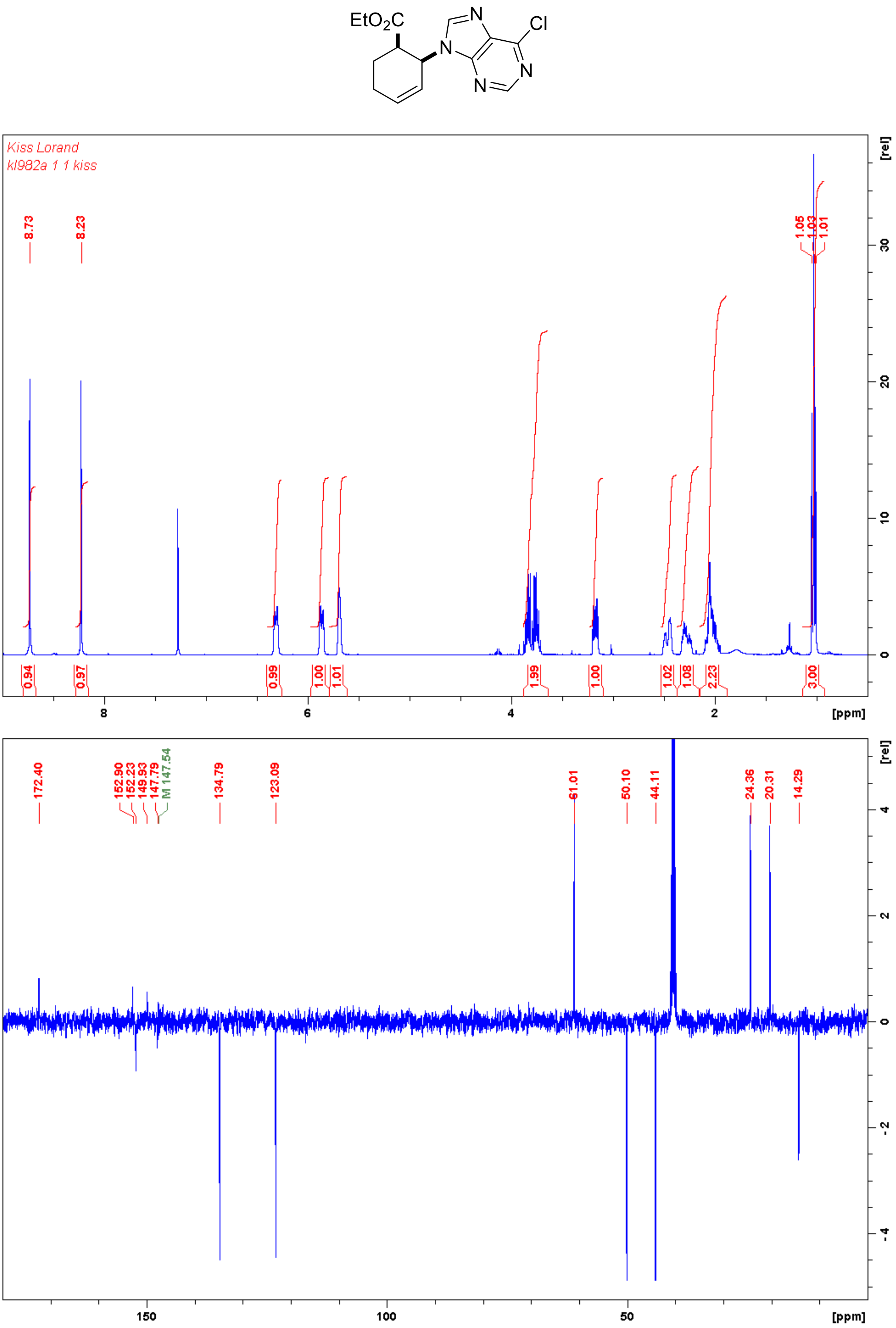

Figure 14. ^{1}H-NMR and ^{13}C-NMR spectra of compound **(±)-35**

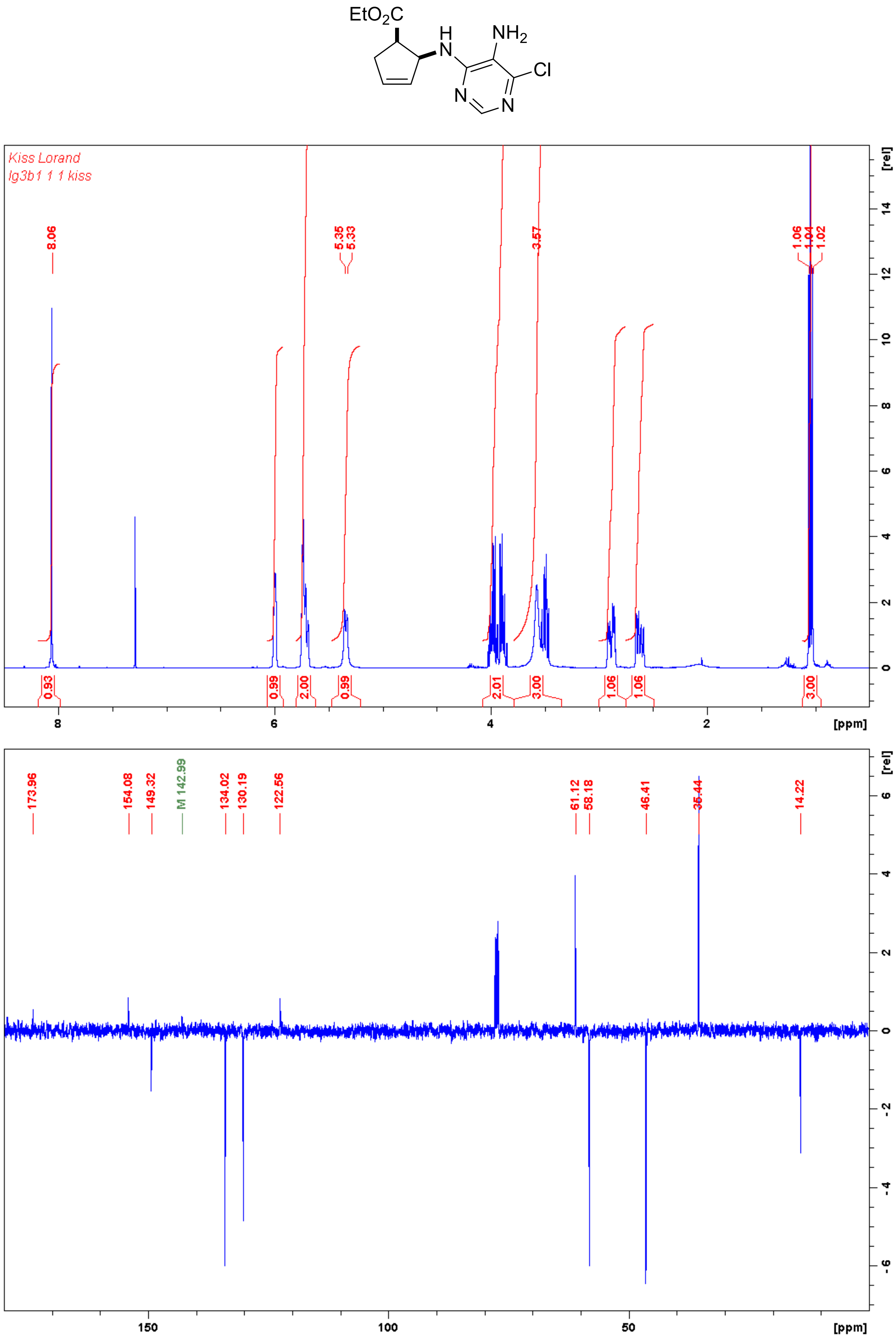

Figure 15. ^{1}H-NMR and ^{13}C-NMR spectra of compound **(±)-38**

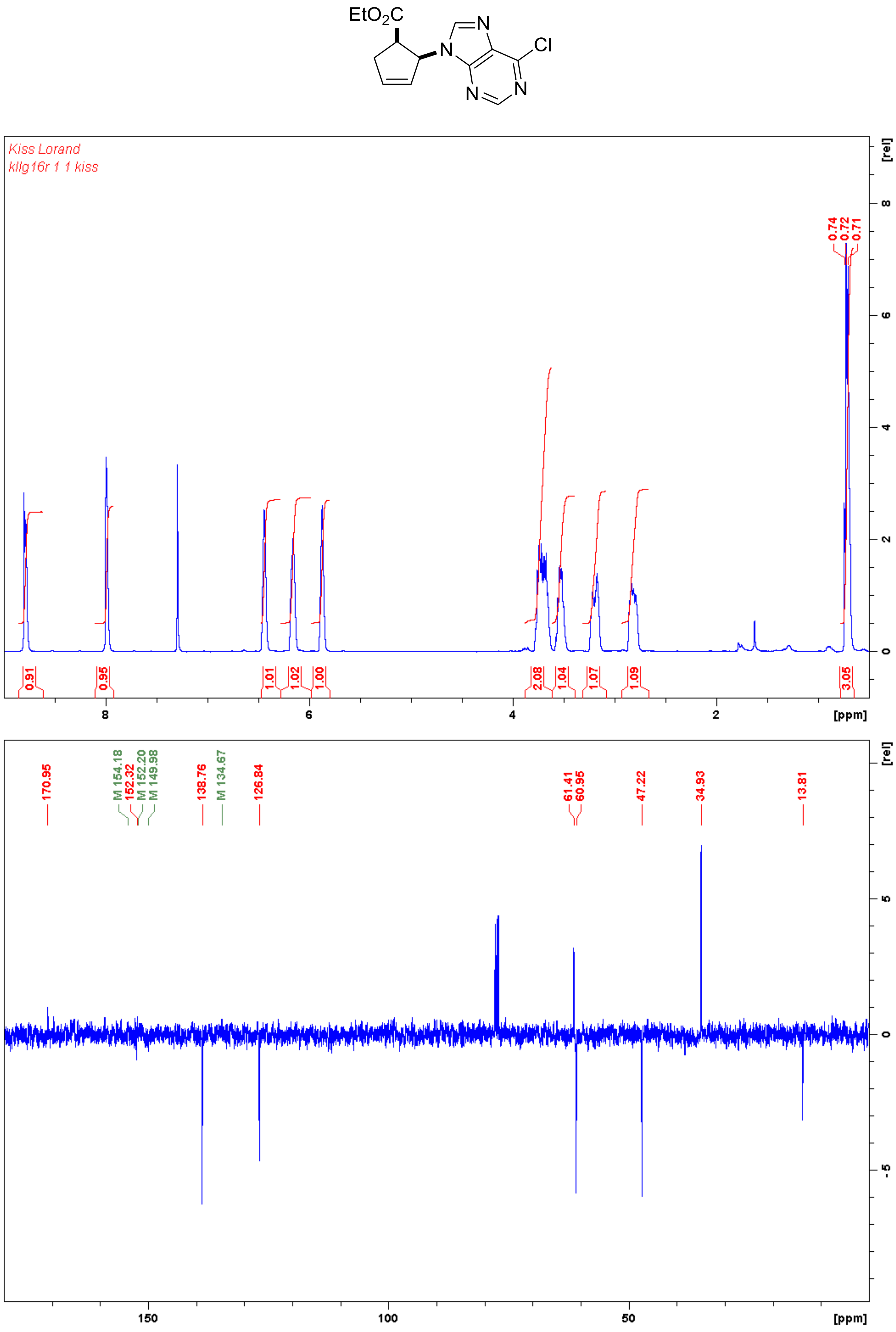

Figure 16. ^{1}H-NMR and ^{13}C-NMR spectra of compound **(±)-39**

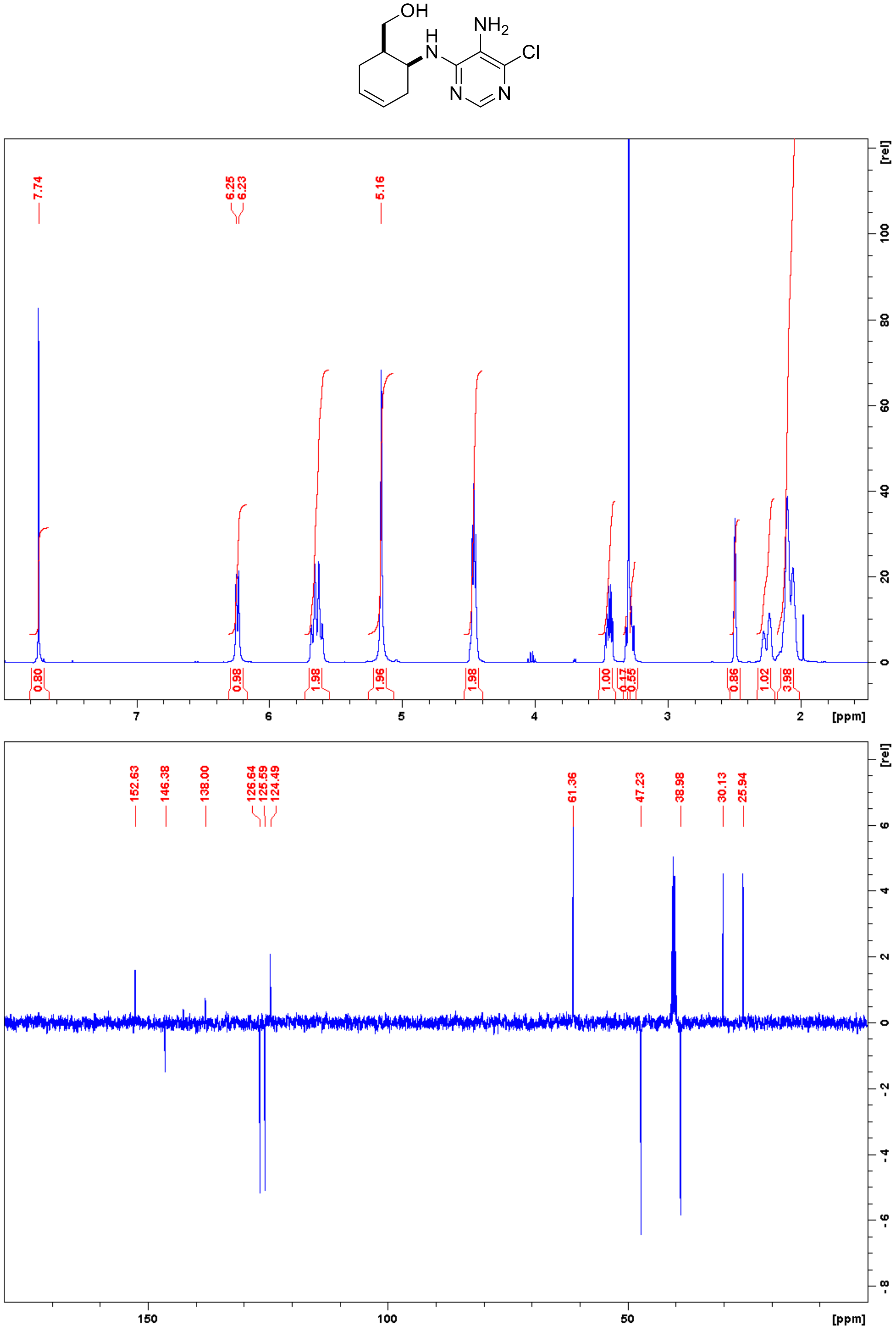

Figure 17. ^{1}H-NMR and ^{13}C-NMR spectra of compound **(±)-42**

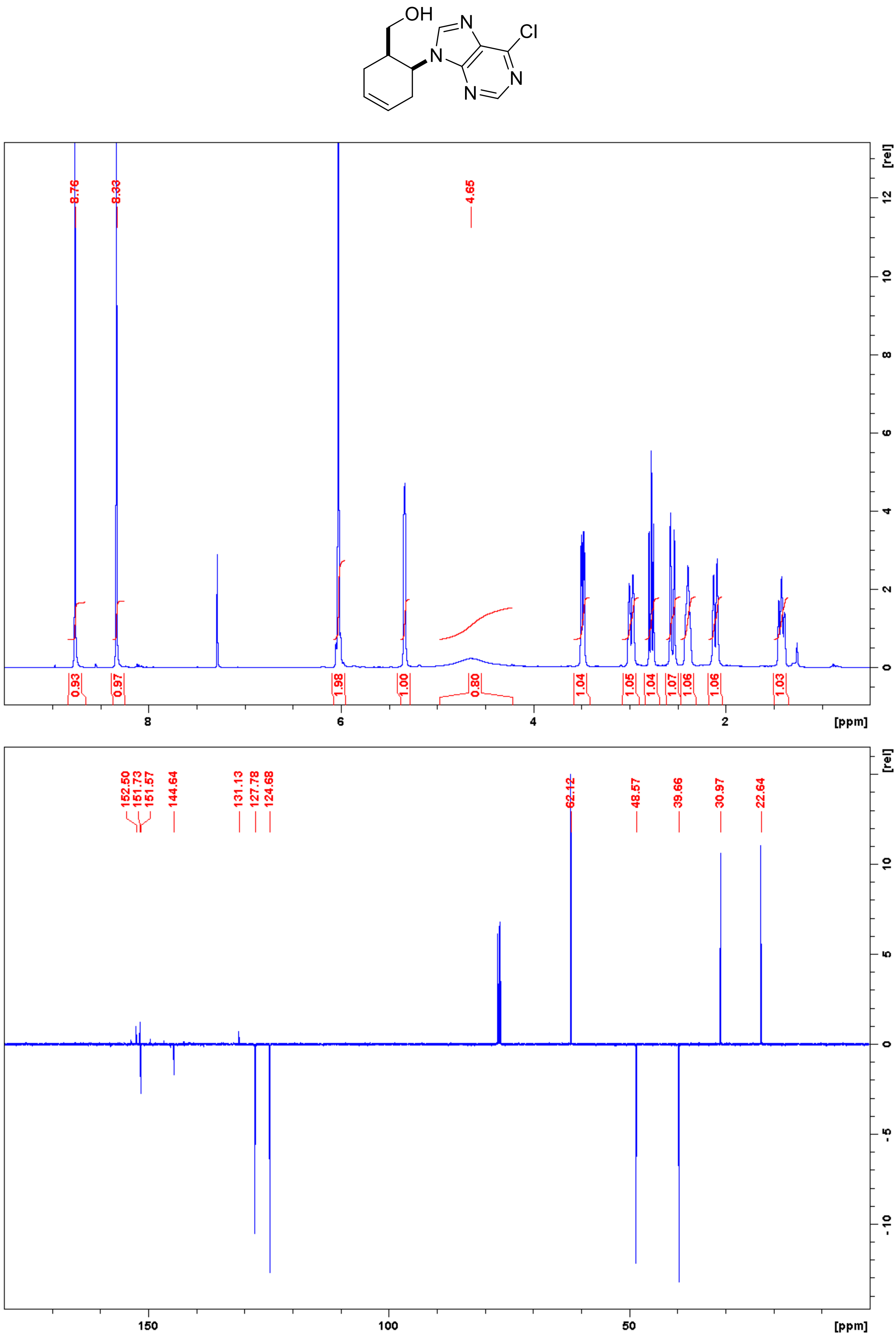

Figure 18. ^{1}H-NMR and ^{13}C-NMR spectra of compound **(±)-43**

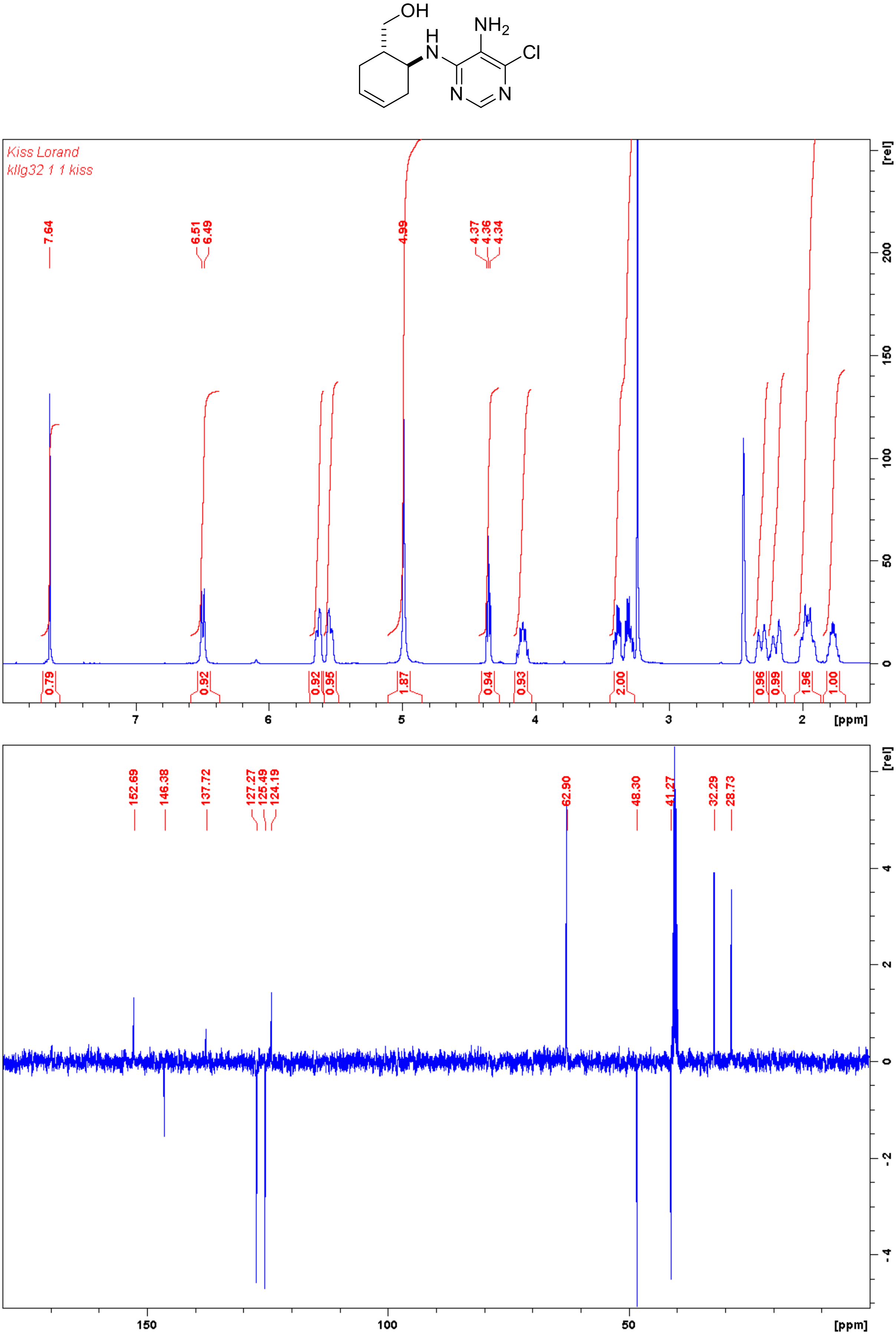

Figure 19. ^{1}H-NMR and ^{13}C-NMR spectra of compound **(±)-46**

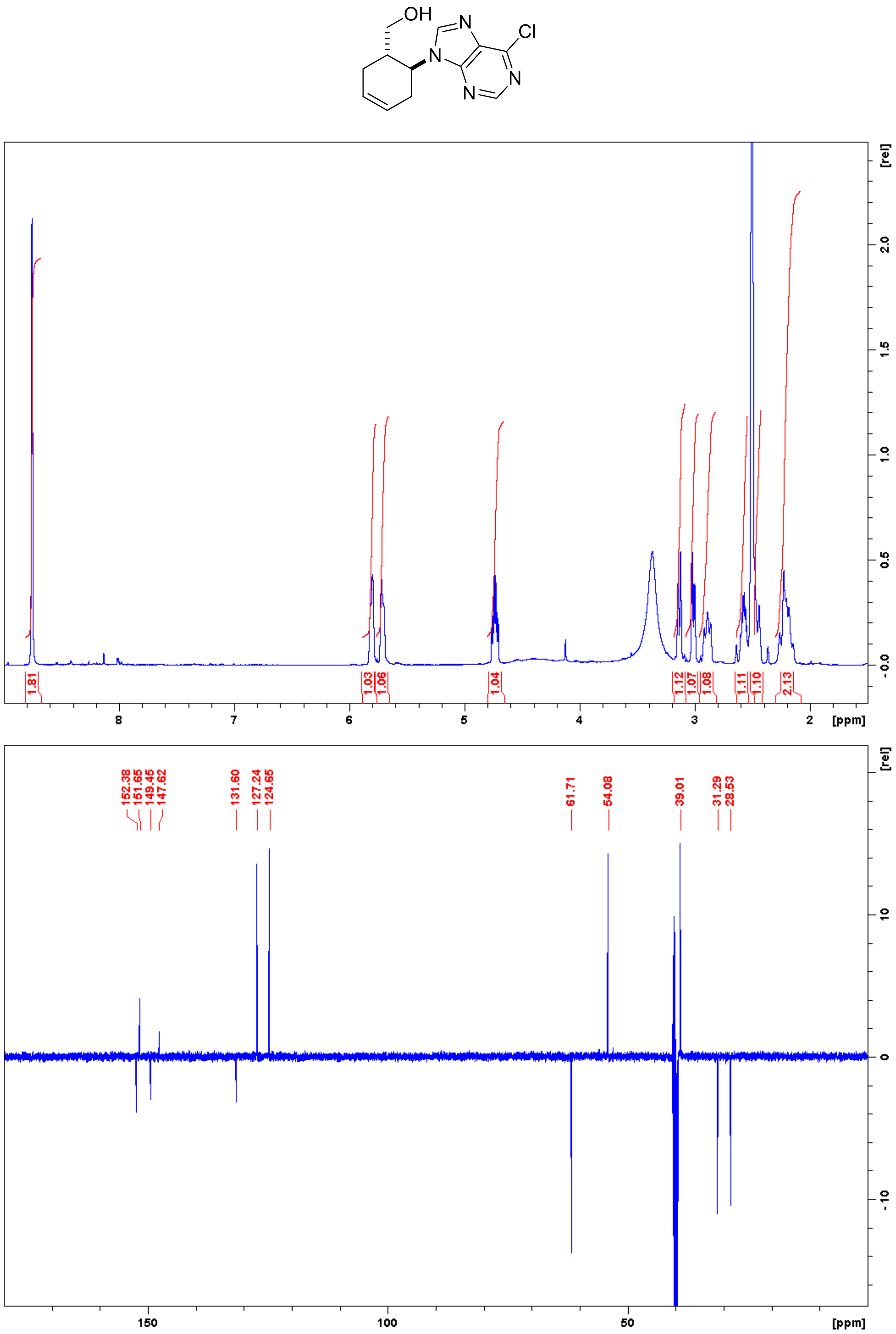

Figure 20. ^{1}H-NMR and ^{13}C-NMR spectra of compound **(±)-47**
